# Gut microbiota and lipopolysaccharide content of the diet influence development of regulatory T cells: studies in germ-free mice

**DOI:** 10.1186/1471-2172-9-65

**Published:** 2008-11-06

**Authors:** Tomas Hrncir, Renata Stepankova, Hana Kozakova, Tomas Hudcovic, Helena Tlaskalova-Hogenova

**Affiliations:** 1 Department of Immunology and Gnotobiology, Institute of Microbiology, Academy of Sciences of the Czech Republic, Prague and Novy Hradek, Czech Republic; 2 Institute of Immunology and Microbiology, 1st Faculty of Medicine, Charles University in Prague, Czech Republic

## Abstract

**Background:**

Mammals are essentially born germ-free but the epithelial surfaces are promptly colonized by astounding numbers of bacteria soon after birth. The most extensive microbial community is harbored by the distal intestine. The gut microbiota outnumber ~10 times the total number of our somatic and germ cells. The host-microbiota relationship has evolved to become mutually beneficial. Studies in germ-free mice have shown that gut microbiota play a crucial role in the development of the immune system. The principal aim of the present study was to elucidate whether the presence of gut microbiota and the quality of a sterile diet containing various amounts of bacterial contaminants, measured by lipopolysaccharide (LPS) content, can influence maturation of the immune system in gnotobiotic mice.

**Results:**

We have found that the presence of gut microbiota and to a lesser extent also the LPS-rich sterile diet drive the expansion of B and T cells in Peyer's patches and mesenteric lymph nodes. The most prominent was the expansion of CD4+ T cells including Foxp3-expressing T cells in mesenteric lymph nodes. Further, we have observed that both the presence of gut microbiota and the LPS-rich sterile diet influence *in vitro *cytokine profile of spleen cells. Both gut microbiota and LPS-rich diet increase the production of interleukin-12 and decrease the production of interleukin-4. In addition, the presence of gut microbiota increases the production of interleukin-10 and interferon-γ.

**Conclusion:**

Our data clearly show that not only live gut microbiota but also microbial components (LPS) contained in sterile diet stimulate the development, expansion and function of the immune system. Finally, we would like to emphasize that the composition of diet should be regularly tested especially in all gnotobiotic models as the LPS content and other microbial components present in the diet may significantly alter the outcome of experiments.

## Background

The mammalian gut harbors a vast and complex microbial community. The human intestinal microflora is estimated to contain 500 to 1000 species and the size of the population is ~10 times greater than the total number of our somatic and germ cells. The role of microbiota in many physiological processes has been demonstrated by using animal models reared under gnotobiological conditions [1-5].

Studies in germ-free (GF) animals have shown that gut microbiota play a crucial role in the development and maturation of the immune system [6-23]. It was demonstrated that the gut-associated lymphoid tissue (GALT), which is the largest immune organ, is immature in GF mice. The content of the lamina propria CD4+ T cells, IgA producing B cells and intraepithelial T cells is reduced in GF animals [8-10,12,13,16,22].

Comparative experiments have also shown that the gene expression profiles of the intestinal epithelial cells are shaped by the presence of gut microbiota and that upregulated genes contribute to secretion of antibacterial molecules at the intestinal surface and the regulation of intestinal angiogenesis [5,15].

The effects of gut microbiota are not only limited to the GALT but systemic immunity is also affected. GF mice have decreased serum immunoglobulin levels and their mesenteric lymph nodes and spleens are smaller and less cellular [11,20,24].

A role of gut microbiota in establishing equilibrium between T_H_1 and T_H_2 immunological responses, which is critical to overall human and animal health, has been postulated [20,25-28]. It is not yet clear whether gut microbiota and microbial components play a role in the development and function of Tregs [29-33] which were recently suggested to be a crucial factor in establishing immunological homeostasis. It has been demonstrated that cells with regulatory function are Foxp3-expressing CD4+ T cells [34-36]. Regulatory T cells (Tregs) suppress activation of the immune system and thereby maintain immune system homeostasis and tolerance to self-antigens and harmless exogenous antigens [34-42]. Depletion or functional abrogation of these cells can cause inflammatory diseases [37-39].

Enormous amount of gut microbiota and their products are in an intimate contact with epithelial surface of the intestinal mucosa. Microbe-associated molecular patterns (MAMPs) present in the intestinal content are sampled mainly by DCs and recognized by their receptors – pattern recognition receptors (PRRs), which include the transmembrane Toll-like receptors (TLRs) [43] and C-type lectin receptors (CLRs) [44], and the cytoplasmic Nod-like receptors (NLRs) [45]. The activated DCs traffic from the intestinal epithelium and Peyer's patches into the mesenteric lymph nodes, where they activate cells of the adaptive immune system.

Gnotobiotic (germ-free) models represent an important tool for unraveling the function of gut microbiota, especially their effects on the mucosal and systemic immunity. Germ-free animals are free of live bacteria but their sterile food contains microbial components and other immunogenic components. To exclude the effects of these components a chemically defined ultrafiltered antigen-free diet was introduced but is rarely used due to technical and financial obstacles [6,24,46-48].

The aim of the present study was to investigate the effects of live gut microbiota and LPS content of the sterile diet as one of the markers of bacterial contamination on the development of the immune system. Our preliminary data have shown that the effect of a low LPS diet (AIN-93G) is negligible in conventional (CV) mice. To address the specific aims of the study we have established three experimental groups different in terms of stimulation with live gut microbiota and LPS: the group of GF mice fed a low LPS diet (AIN-93G), the group of GF mice fed a LPS-rich diet (ST1) and the fully stimulated group of CV mice (colonized by gut microbiota and fed a LPS-rich diet).

To analyze the effects of gut microbiota and LPS content of the sterile diet on the maturation of the immune system, the weight, cellularity and cellular composition of lymphoid organs were compared using the above-mentioned experimental groups. In addition to the immunophenotypic analysis of major lymphocyte populations, we have focused on the subpopulation of Foxp3-expressing T cells. The study was complemented by determining *in vitro *proliferative and cytokine response of spleen cells to LPS and concanavalin (ConA).

## Results

### Purified diet may have almost 100 times lower LPS content than a grain-based diet

**Figure 1:**
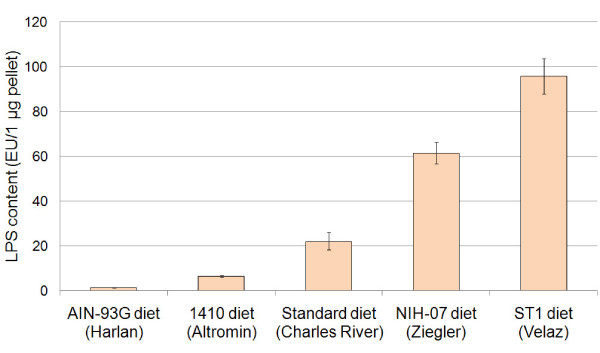
**Comparison of LPS content of mouse pelleted diets**. The concentration of LPS was measured to determine the load of microbiota-derived components in mouse diets. The pellets were ground, sonicated in non-pyrogenic water and filtered. LPS concentration in the filtrate was analyzed using the Chromogenic Limulus Amebocyte Lysate (LAL) Test (Cambrex, USA) and is expressed as endotoxin units (EU) per 1 μg of a diet. Results represent the mean (± SE) of four measurements.

We have decided to use LPS concentration as a measure of overall bacterial contamination in diets. To study the effect of LPS content of the sterile diet on the development of the immune system we had to first identify the diets with both very low and very high LPS content. The concentration of LPS was measured by chromogenic LAL test and expressed in endotoxin units (EU) per μg of pellet. The diets sorted by LPS concentration from the lowest to the highest were AIN-93G diet (Harlan, USA), 1410 diet (Altromin, Germany), Standard diet (Charles River, USA), NIH-07 diet (Zeigler, USA) and ST1 diet (Velaz, Czech Republic). In our experiments we used AIN-93G diet, which is a purified diet, and ST1 diet, which is a grain-based diet. These diets have been selected on the basis of maximum difference in the LPS content (Fig. 1).

### Gut microbiota and the LPS-rich sterile diet increase the weight and cellularity of lymphoid organs

**Figure 2:**
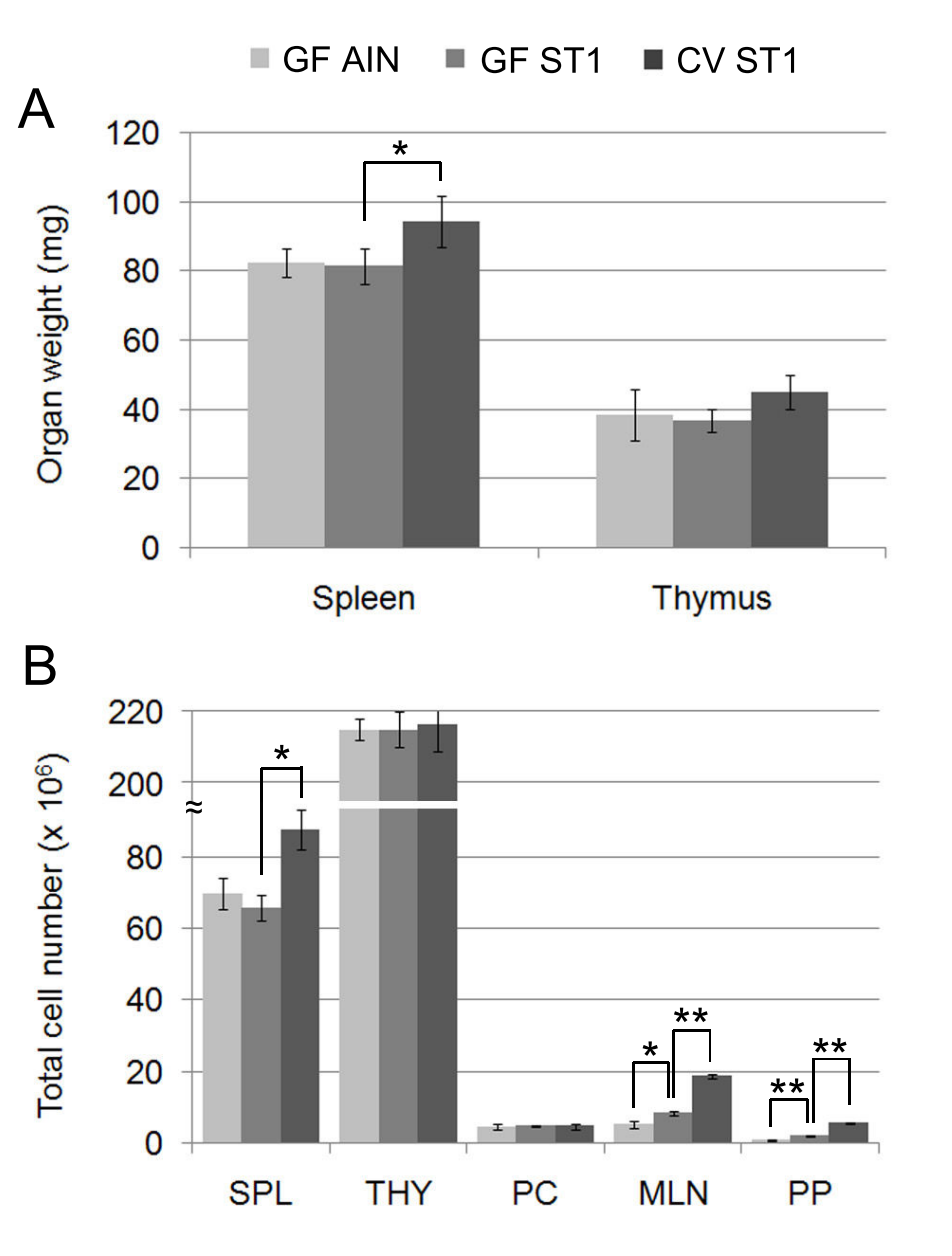
**The effect of gut microbiota and LPS-rich sterile diet on the weight and cellularity of lymphoid organs**. (A) Germ-free mice have smaller spleens compared to conventional mice. (B) Gut microbiota and the LPS-rich diet drive the cellular expansion in MLNs and PPs. Results represent the mean (± SE) of at least 10 mice/group. Statistical analyses were performed using one-way analysis of variance (ANOVA) and a post-hoc comparison test (Tukey-Kramer). * indicates p < 0.05 and ** indicates p < 0.01.

We did not detect any significant difference in the weight and cellularity of thymus isolated from CV or GF mice fed the low LPS diet (AIN-93G) or LPS-rich diet (ST1). However, we observed a decreased weight and cellularity of spleens from GF mice fed either diet compared to spleens from CV mice (Fig. 2A and 2B). The peritoneal cell number was not affected by the LPS content of the sterile diet or by gut microbiota. In contrast, the overall MLN and PP cell numbers increased in GF mice fed the LPS-rich diet (ST1) compared to GF mice fed the low LPS diet (AIN-93G) and the cell numbers further increased in the group of CV mice (Fig. 2B).

### Gut microbiota and the LPS-rich diet decreases the proportion of CD19+ B cells in MLNs

**Figure 3:**
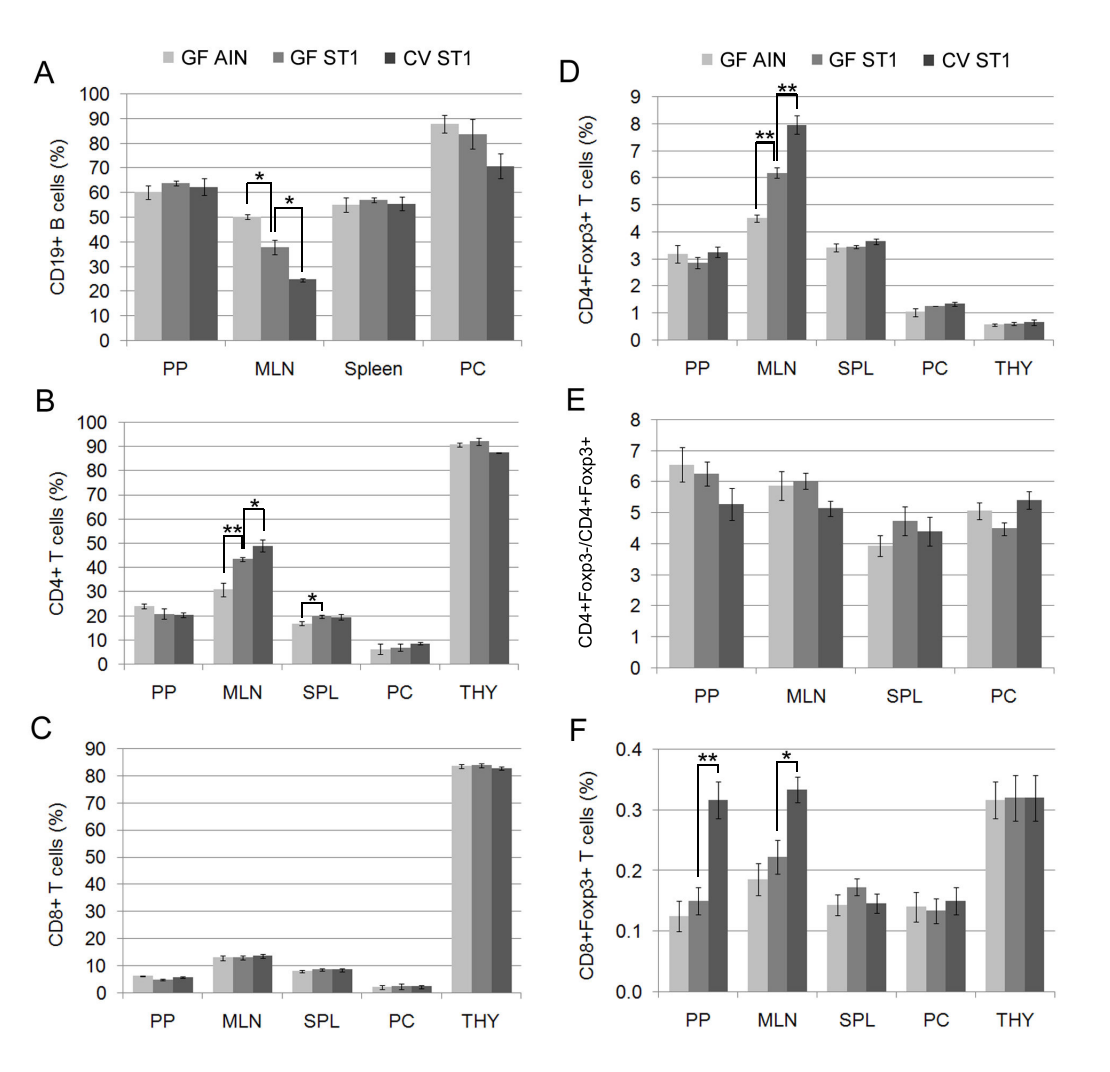
**FACS analysis of lymphocyte subpopulations illustrates the stimulating effect of gut microbiota and LPS-rich sterile diet**. (A) Both gut microbiota and the LPS-rich diet decrease the proportion of CD19+ B cells in MLNs. (B) LPS-rich diet induces the expansion of CD4+ T cells in MLNs and spleen. (C) Microbial stimulation does not affect the proportion of CD8+ T cells. (D) Both gut microbiota and the LPS-rich sterile diet induce the expansion of Foxp3-expressing CD4+ T cells in MLNs. (E) The ratio of CD4+Foxp3- T cells to CD4+Foxp3+ T cells remains constant in all lymphoid organs. (F) Gut microbiota stimulate the expansion of Foxp3-expressing CD8+ T cells in PPs and MLNs. Results represent the mean (± SE) of at least 10 mice/group. Statistical analyses were performed using one-way analysis of variance (ANOVA) and a post-hoc comparison test (Tukey-Kramer). * indicates p < 0.05 and ** indicates p < 0.01.

We found that MLNs of GF mice fed the low LPS diet (AIN-93G) have a higher proportion of CD19+ B cells than GF mice fed the LPS-rich diet (ST1). In addition, the group of GF mice fed the LPS-rich diet (ST1) had a higher proportion of CD19+ B cells in MLNs than the group of CV mice (Fig. 3A). However, we would like to emphasize that the presence of gut microbiota increases the absolute numbers of CD19+ B cells in PPs, MLNs and spleen and that the LPS-rich diet increases the absolute number in PPs (Table 1). The observed discrepancy between the proportion and total number of CD19+ B cells in MLNs is mainly caused by the vigorous expansion of MLN CD4+ T cells. The proportion of CD19+ B cells in Peyer's patches, spleen and peritoneal cells remains constant irrespective of the degree of microbial stimulation.

**Table 1:** FACS analysis of lymphocyte subpopulations in conventional and germ-free Balb/c mice fed either a low LPS diet (AIN-93G) or a LPS-rich diet (ST1)

		Number of positive lymphocytes (× 10^6^)		
Lymphoid organ/tissue	Subpopulation	CV ST1	GF ST1	GF AIN
Peyer's patches	Total	4.69 ± 0.16	1.57 ± 0.19	0.67 ± 0.09
	CD19+	2.93 ± 0.10	1.00 ± 0.12	0.40 ± 0.05
	CD3+	1.38 ± 0.05	0.44 ± 0.05	0.21 ± 0.03
	CD4+	0.95 ± 0.03	0.33 ± 0.04	0.16 ± 0.02
	CD8+	0.27 ± 0.01	0.08 ± 0.01	0.04 ± 0.01
	CD4+Foxp3+	0.15 ± 0.01	0.04 ± 0.01	0.02 ± 0.00
	CD8+Foxp3+	0.02 ± 0.00	0.00 ± 0.00	0.00 ± 0.00
Mesenteric lymph nodes	Total	17.81 ± 0.69	7.82 ± 0.57	4.96 ± 0.96
	CD19+	4.39 ± 0.17	2.96 ± 0.21	2.48 ± 0.48
	CD3+	12.10 ± 0.47	4.70 ± 0.34	2.36 ± 0.46
	CD4+	8.70 ± 0.34	3.40 ± 0.25	1.53 ± 0.30
	CD8+	2.41 ± 0.09	1.03 ± 0.07	0.64 ± 0.12
	CD4+Foxp3+	1.42 ± 0.05	0.48 ± 0.04	0.22 ± 0.04
	CD8+Foxp3+	0.06 ± 0.00	0.02 ± 0.00	0.01 ± 0.00
Spleen	Total	83.14 ± 5.28	62.48 ± 3.46	66.21 ± 4.12
	CD19+	46.14 ± 2.93	35.59 ± 1.97	36.44 ± 2.27
	CD3+	24.74 ± 1.57	20.54 ± 1.14	16.93 ± 1.05
	CD4+	16.32 ± 1.04	12.35 ± 0.68	11.18 ± 0.70
	CD8+	7.00 ± 0.44	5.36 ± 0.30	5.31 ± 0.33
	CD4+Foxp3+	3.03 ± 0.19	2.16 ± 0.12	2.27 ± 0.14
	CD8+Foxp3+	0.12 ± 0.01	0.12 ± 0.01	0.11 ± 0.01
Peritoneal cells	Total	2.55 ± 0.41	2.65 ± 0.06	2.50 ± 0.35
	CD19+	1.80 ± 0.29	2.22 ± 0.05	2.19 ± 0.30
	CD3+	0.11 ± 0.02	0.08 ± 0.00	0.07 ± 0.01
	CD4+	0.22 ± 0.04	0.18 ± 0.00	0.16 ± 0.02
	CD8+	0.06 ± 0.01	0.06 ± 0.00	0.05 ± 0.01
	CD4+Foxp3+	0.03 ± 0.01	0.03 ± 0.00	0.03 ± 0.00
	CD8+Foxp3+	0.00 ± 0.00	0.00 ± 0.00	0.00 ± 0.00
Thymus	Total	207.03 ± 9.23	205.76 ± 8.46	205.76 ± 7.98
	CD4-CD8-	8.70 ± 0.30	10.72 ± 0.50	11.87 ± 0.20
	CD4+CD8+	155.27 ± 8.13	151.59 ± 7.26	146.52 ± 7.18
	CD4+CD8-	30.23 ± 1.82	28.81 ± 1.72	31.39 ± 2.39
	CD8+CD4-	13.04 ± 0.67	11.93 ± 0.57	9.88 ± 0.57
	CD4+Foxp3+	1.35 ± 0.05	1.25 ± 0.03	1.15 ± 0.02
	CD8+Foxp3+	0.66 ± 0.02	0.66 ± 0.02	0.65 ± 0.01

The single cell suspensions were stained with the monoclonal antibodies as described under "Methods" and analyzed by FACS. PP and MLN cell numbers represent the overall cell pool isolated from one mouse. Data is a summary of at least four independent experiments (at least 10 mice/group) and values represent the mean ± SE.

### Gut microbiota and the LPS-rich diet stimulate the expansion of CD4+ T cells

To determine the role of gut microbiota and LPS content of the diet in the development of major T cell subpopulations, including Foxp3-expressing T cells, we have isolated cells from Peyer's patches, MLNs, spleen, thymus and peritoneum of CV and GF mice fed either the low LPS diet (AIN-93G) or the LPS-rich diet (ST1). The distinct cell surface and intracellular markers were analyzed by flow cytometry. We observed that the stimulating effect of LPS-rich sterile diet leads to the expansion of CD4+ T cells in MLNs and spleen. The presence of gut microbiota further increased the proportion of CD4+ T cells in MLNs but not in spleen where the LPS-rich diet had already increased the proportion of CD4+ T cells to the level found in CV mice. The proportion of CD4+ T cells in Peyer's patches, thymus and peritoneal cells remains constant regardless of the level of microbial stimulation (Fig. 3B).

### Neither gut microbiota nor the LPS-rich diet affects the proportion of CD8+ T cells in lymphoid organs

The presence of gut microbiota has no effect on the proportion of CD8+ T cells in spleen, as previously described [48]. In addition, we show that the proportion of CD8+ T cells is independent of gut colonization also in other lymphoid organs including MLNs, Peyer's patches, thymus and peritoneal cells. In accordance with these findings we did not observe any effect of LPS content of the diet on the proportion of CD8+ T cells in lymphoid organs of GF mice (Fig. 3C).

### Both gut microbiota and the LPS-rich diet drive the expansion of Foxp3-expressing CD4+ T cells in MLNs

To investigate the effect of gut microbiota and LPS content of the sterile diet on the development of CD4+Foxp3+ T cells we analyzed their proportion in Peyer's patches, MLNs, spleen, thymus and peritoneal cells in CV mice and GF mice fed either the low LPS diet (AIN-93G) or LPS-rich diet (ST1). We found that both gut microbiota and the LPS-rich diet drive the expansion of CD4+Foxp3+ T cells in MLNs (Fig. 3D). However, the proportion of CD4+Foxp3+ T cells in other lymphoid organs was not affected. We would like to stress that the CD4+Foxp3-/CD4+Foxp3+ ratio remains constant in all lymphoid organs including MLNs and is not influenced during the lymphocyte expansion driven by gut microbiota or LPS (Fig. 3E).

### Gut microbiota stimulate the expansion of Foxp3-expressing CD8+ T cells in Peyer's patches and MLNs

We observed a stimulating effect of gut microbiota on CD8+Foxp3+ T cells. The proportion of CD8+Foxp3+ T cells increased in Peyer's patches and MLNs. The effect of LPS-rich diet on the expansion of CD8+Foxp3+ T cells in all lymphoid organs of GF mice was not significant (Fig. 3F).

### *In vitro *proliferative response of spleen cells is not influenced by gut microbiota or LPS content of the sterile diet

**Figure 4:**
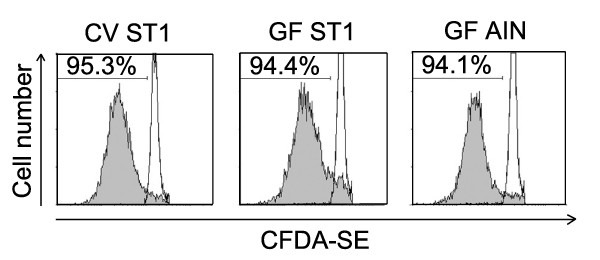
***In vitro *proliferative response of spleen cells is not influenced by gut microbiota or LPS-rich sterile diet**. Spleen cells isolated from CV mice and GF mice fed either diet were stained with CFSE fluorescein and stimulated with ConA. After 72 h the cells were analyzed by FACS. Histograms in this figure show the CFSE staining profile of lymphocytes negative for CD19 antigen (B cell marker). Proliferation was measured as the percentage of cells showing decreased staining intensity of CFSE compared to the intensity of the CFSE^bright ^population. Open histograms represent unstimulated control cells. The presented data are from a representative experiment. Each experiment was repeated at least three times with similar results.

Spleen cells isolated from CV mice and GF mice fed either the low LPS diet (AIN-93G) or LPS-rich diet (ST1) were stained with CFSE fluorescein and stimulated with concanavalin A (ConA). After 72 h the CFSE staining profile of spleen cells negative for CD19 antigen (B cell marker) was analyzed by flow cytometry. No significant difference was found between the groups of CV and GF mice fed either diet (Fig. 4). Thus we conclude that neither gut microbiota nor LPS content of the sterile diet influences non-specific proliferative response of spleen cells *in vitro*.

### Both gut microbiota and the LPS-rich sterile diet influence a spleen cell cytokine profile

**Figure 5:**
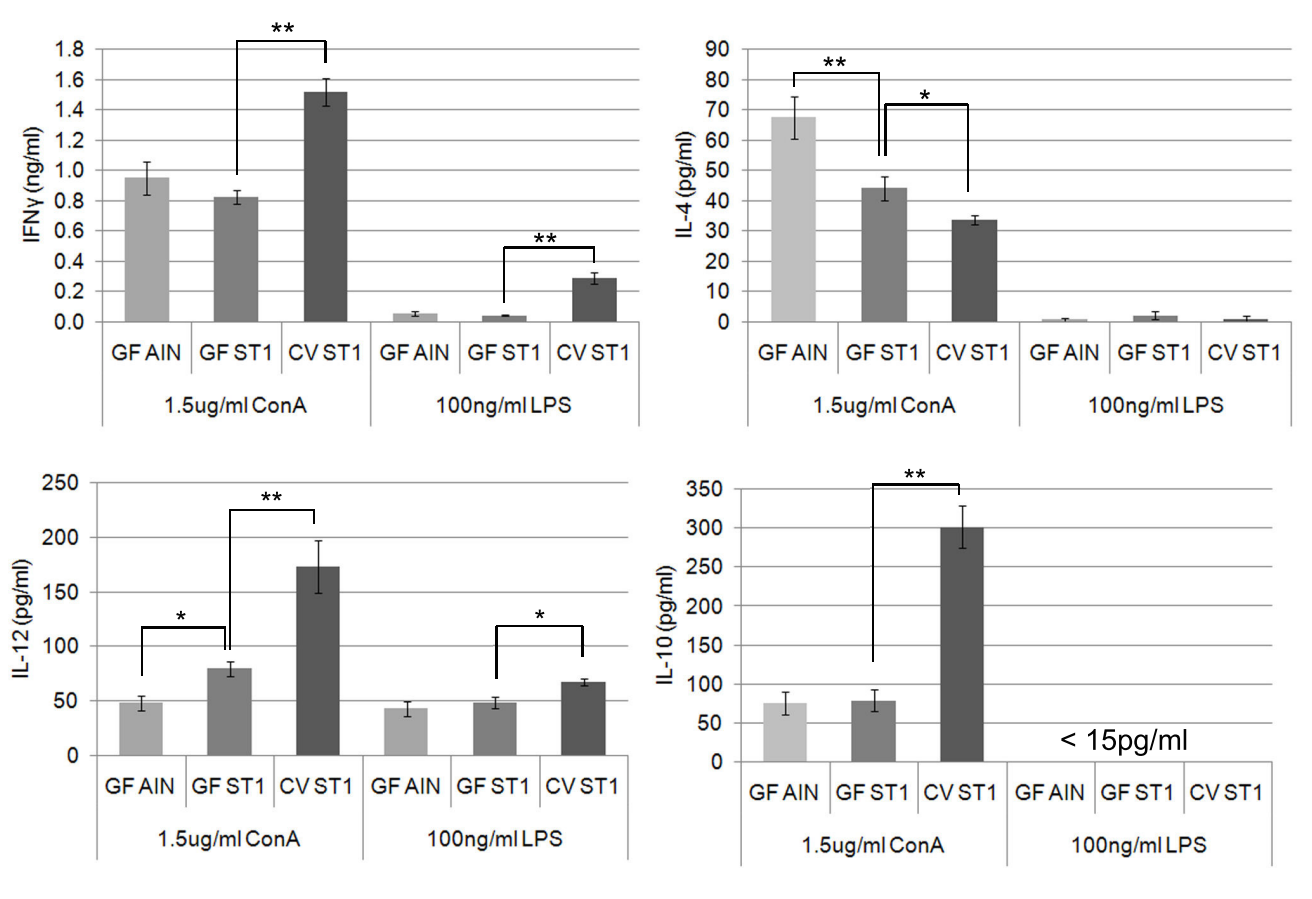
**Gut microbiota and the LPS-rich diet influence *in vitro *cytokine response of spleen cells**. Spleen cells from CV and GF mice fed the low LPS diet (AIN-93G) or LPS-rich diet (ST1) were stimulated with ConA or LPS for 48 h. The production of IFNγ, IL-4, IL-10 and IL-12 cytokines was determined by Luminex analyzer. Results represent the mean (± SE) of at least 6 mice/group. Statistical analyses were performed using one-way analysis of variance (ANOVA) and a post-hoc comparison test (Tukey-Kramer). * indicates p < 0.05 and ** indicates p < 0.01.

To characterize the effect of gut microbiota and LPS content of the sterile diet on a cytokine response, spleen cells were stimulated with ConA and LPS. We have observed that both gut microbiota and the LPS-rich sterile diet influence *in vitro *cytokine profile of spleen cells. Both gut microbiota and LPS-rich diet increase the production of interleukin-12 and decrease the production of interleukin-4 after ConA stimulation. In addition, the presence of gut microbiota increases the production of interferon-γ and an anti-inflammatory cytokine interleukin-10. We have also found that the presence of gut microbiota increases the production of interferon γ and interleukin-12 in response to LPS stimulation (Fig. 5).

## Discussion

### LPS-driven lymphocyte expansion in PPs and MLNs of germ-free mice

It is generally accepted that live gut microbiota are essential for the development and maturation of the mammalian immune system [10,11,14,17,18,20,21]. Multiple studies revealed that animals kept under germ-free conditions have reduced cellular components of mucosal and systemic immunity. GF mice have decreased frequency of DCs, CD4+ T cells, IgA producing B cells and intraepithelial T cells in gut lamina propria [12,20,22,23,49]. PPs and MLNs are smaller, less cellular (lower numbers of B and T cells) and do not have germinal centers [1,2,7,11,47]. Spleens of GF mice are also smaller, less cellular and the proportion of CD4+ T cells is reduced [20]. Our studies confirm the important role played by gut microbiota. In addition, we show that microbiota-derived components present in the sterile diet stimulate the development of the immune system even in the absence of live gut microbiota. In the present study, we show that LPS-rich sterile diet partially corrects the profound immunological deficiencies found in GF mice. We demonstrate that LPS-rich diet stimulates the expansion of all major lymphocyte subpopulations in GF MLNs and PPs, including CD19+ B cells, CD8+ T cells and CD4+ T cells. The proportions of CD19+ B cells, CD8+ T cells, and CD4+ T cells in PPs of GF mice remain constant during the LPS-driven expansion. In contrast, we observed a significant increase in the proportion of CD4+ T cells at the expense of CD19+ B cells in MLNs of GF mice. Our observation of the significant increase in the proportion of CD4+ T cells in spleen of GF mice fed LPS-rich diet is in line with recent studies showing that monocolonization of germ-free animals with *Bacteroides fragilis *results in CD4+ T cell expansion [20].

### The effect of gut microbiota and LPS-rich diet on the development of Foxp3-expressing T cells

Regulatory T cells are a component of the immune system that suppresses activation of other immune cells and thus maintains immune system homeostasis. The latest research suggests that Tregs are best defined by the expression of the transcription factor Foxp3 [34,35,40]. The large majority of Foxp3-expressing Tregs is found within CD4+ helper T cell population and expresses high levels of the interleukin-2 receptor alpha chain (CD25). Regulatory T cells comprise about 5–10% of the mature CD4+ helper T cell subpopulation in mice. Mutations in the gene encoding Foxp3 result in the development of overwhelming systemic autoimmunity in the first year of life in both humans and mice.

It is still controversial whether gut microbiota and microbiota-derived components play a role in the development and maturation of Tregs. In the transfer model of colitis developing in CD4+CD45RB^high ^T cell reconstituted immune-deficient SCID mice we have shown that the presence of normal gut microbiota enhances a functional potency of the Treg population. The inhibitory activity of CD4+CD45RB^low ^T cells from GF mice was significantly impaired compared to the population isolated from specific-pathogen free mice [37]. It has been recently reported that gut microbiota are crucial for the generation and expansion of Tregs [31]. Ostman et al. reported that CD25+ Tregs from GF mice are less effective in suppressing proliferation of responder CD4+CD25- T cells. However, they did not find any difference in the proportion of CD4+Foxp3+ T cells between CV and GF mice. The only deficit of CD4+Foxp3+ T cells in GF mice was detected in the liver-draining celiac lymph nodes [32]. Paradoxically, it was reported that CD25+ Tregs from GF mice are as suppressive and protective as those from CV mice [29,30] and Min et al. recently reported that peptide antigens derived from intestinal microorganisms are not essential for the generation, in vivo proliferation or suppressive activity of Tregs [33].

Our results are consistent with the results of Strauch et al. and Ostman et al., which demonstrate the important role of gut microbiota in the development and function of Tregs. We show that both live gut microbiota and LPS-rich sterile diet expand the absolute cell numbers of Foxp3-expressing CD4+ T cells in MLNs and PPs (Table 1). In addition, we observed that the stimulating effect of both gut microbiota and LPS-rich sterile diet significantly increased the proportion of CD4+Foxp3+ T cells in MLNs.

### Gut microbiota and inflammatory diseases

Increase in hygienic standards associated with a lack of undefined infectious stimuli mainly during the early postnatal period was regarded to be responsible for increasing prevalence of chronic inflammatory diseases in developed countries – the so called "hygiene hypothesis" was formulated [28].

Based on epidemiological studies it was suggested that the composition of gut microbiota could affect the susceptibility to the development of inflammatory diseases including allergic and autoimmune diseases [50-52]. The major recently proposed mechanism explaining the "hygiene hypothesis" is that the developing immune system must receive sufficient stimuli in order to adequately develop Tregs, or it will be more susceptible to autoimmune and allergic diseases, because of insufficiently repressed effector T_H_1 and T_H_2 responses, respectively [52]. Our data are in line with the proposed mechanism as we have found that both gut microbiota as well as LPS-rich diet drive the expansion of CD4+Foxp3+ T cells in MLNs. In addition, we observed a stimulating effect of gut microbiota on IL-10 production.

The role of Tregs in the development of chronic inflammatory diseases and its relation to microbiota composition is currently the subject of our research performed in gnotobiotic animal models.

### How microbiota-derived components influence the maturation of the immune system

Several mechanisms by which gut microbiota and their components may influence the development of the immune system have been proposed. According to the current knowledge the nature of TLR, CLR and NLR ligands selectively determines the cytokine production by DCs and thus modulates T-cell differentiation.

Gut intraluminal antigens are sampled by DCs in the Peyer's patches and the intestinal epithelium and carried to the MLNs via the afferent lymphatics. In the MLNs the DCs induce T-cell activation and differentiation. The DCs are activated through the recognition of microbial components, such as LPS. However, other activating components of the intestinal content including fatty acids (recognized by TLR4 receptor) [53], beta-glucan (recognized by dectin-1 receptor) [54], wheat [55] and other molecules may be involved in the initiation of immune responses. The activation of DCs leads to the production of cytokines and expression of co-stimulatory molecules. The presentation of processed antigens bound to MHC class II results in the activation and differentiation of T cells. The cytokines secreted by activated DCs play a critical role in T-cell differentiation. The pivotal cytokines that control T-cell differentiation are IFNγ and IL-12 (T_H_1), IL-4 (T_H_2) and TGF-β and IL-6 (T_H_17) and TGF-β (Tregs). The activated CD4+ T cells then migrate to effector tissues where they help to orchestrate the immune responses.

MLNs serve as a bridge between innate and adaptive immunity and are the key site for the induction of mucosal tolerance to intestinal antigens [56]. Our data show that the stimulating effect of gut microbiota and microbiota-derived components is essential for the maturation of CD4+ T cell subpopulations including Foxp3-expressing T cells in MLNs.

## Conclusion

In conclusion, our results demonstrate that the presence of gut microbiota and to a lesser extent also the LPS-rich sterile diet drive the expansion of B and T cells in Peyer's patches and mesenteric lymph nodes. The most prominent was the expansion of CD4+ T cells including Foxp3-expressing T cells in mesenteric lymph nodes. Further, we have observed that both the presence of gut microbiota and the LPS-rich sterile diet influence *in vitro *cytokine profile of spleen cells. Both gut microbiota and LPS-rich diet increase the production of interleukin-12 and decrease the production of interleukin-4. In addition, the presence of gut microbiota increases the production of interleukin-10 and interferon-γ. Finally, we would like to emphasize that the content of microbial components in sterile diets has a significant effect on the development and function of the immune system under germ-free conditions and thus the quality of diet should be tested in all gnotobiotic models.

## Methods

### Mice

Both germ-free (GF) and conventional (CV) Balb/c mice were maintained on a sterile experimental diet for at least two generations. After weaning the mice were fed a sterile experimental diet ad libitum and used in experiments at the age of 8–10 weeks.

Long-term colonies of germ-free Balb/c mice have been established using the rederivation by a Caesarean section and maintained in flexible plastic isolators. Fecal samples from GF mice and swabs from the inner surface of the isolators were cultured under both aerobic and anaerobic conditions on a weekly basis and prior to the experiment to verify continued sterility of the colony. The conventional Balb/c mice were regularly checked for the absence of potential pathogens according to an internationally established standard (FELASA). All the experiments were performed in the Department of Immunology and Gnotobiology of the Institute of Microbiology of the AS CR. All the researchers who handled the animals have been certified by the Central Committee for Animal Welfare. The Institute of Microbiology is authorized by the Central Committee for Animal Welfare to carry out experiments on laboratory animals. The local ethical guidelines are in compliance with Directive 86/609/EEC on the protection of animals used for experimental and other scientific purposes and Recommendation 2007/526/EC of the European Commission.

### Determination of LPS content of mouse feed pellets

We have tested all the pelleted diets which are commonly used in our animal facility. Namely AIN-93G diet (Harlan, USA), 1430 diet with gluten-free modification (Altromin, Germany), Charles River's standard diet (Charles River, USA), rodent NIH-07 22.5–5 diet (Ziegler, USA) and ST1 diet (Velaz, Czech Republic). To determine the LPS content of mouse diets, the pellets were ground, sonicated in non-pyrogenic water and filtered. LPS concentration in the filtrate was measured using the Chromogenic Limulus Amebocyte Lysate (LAL) Test (Cambrex, USA) and expressed as endotoxin units (EU) per 1 μg of a diet.

### Diets

Mice were fed ad libitum with either a purified diet (AIN-93G, Harlan) or a grain-based diet (ST1, Velaz). Both diets were sterilized by irradiation. The AIN-93G diet is a growth diet for rodents recommended by the American Institute of Nutrition. It is based mainly on purified ingredients, such as corn starch, vitamin free casein, maltodextrin, sucrose, soybean oil and powdered cellulose supplemented with mineral and vitamin mix. The ST1 diet is a grain-based diet which is based mainly on wheat, oat, corn, wheat flour, snail clover fodder, soya pollard and scrap cake. The detailed composition of a purified diet (AIN-93G) and a grain-based diet (ST1) is presented in Tables D1 and D2, respectively (Additional files 1 and 2, respectively). Both experimental diets were nutritionally adequate and animal growth curves were comparable. The AIN-93G diet has an almost 100 times lower content of LPS than ST1 diet.

### Preparation of cell suspensions

The organs were cut with scissors, squeezed with a syringe plunger and filtered through a 70 μm cell strainer (BD Falcon, USA). Red blood cells in spleen cell suspensions were lysed with ACK lysing buffer (0.15 M NH_4_Cl, 10 mM KHCO_3_, 0.1 M Na_2_EDTA, pH 7.3) for 5 min at room temperature. All the cells were washed twice and resuspended in complete RPMI medium (RPMI-1640 medium containing 10% fetal bovine serum (FBS), 2 mM L-glutamine, 50 μM 2-mercaptoethanol, 100 U/ml penicillin and 100 μg/ml streptomycin sulphate) or FACS buffer (PBS containing 0.1% NaN_3 _and 0.5% FBS). To harvest resident peritoneal cells, 10 ml complete RPMI medium per mouse was injected into the peritoneal cavity. Collected peritoneal lavage fluid was centrifuged and then resuspended in harvest medium. The cells were counted and adjusted to appropriate cell concentration.

### Flow cytometry and intracellular cytokine staining

Phenotypic analysis of cells isolated from spleen, thymus, MLNs, PPs and peritoneal cavity was performed by flow cytometry. The following mAb with matching isotype controls were used: FITC-conjugated anti-mouse CD3e (BD Pharmingen, USA), PE-conjugated anti-mouse CD19 (BD Pharmingen, USA), FITC-conjugated anti-mouse CD4 (BD Pharmingen, USA), PE-conjugated anti-mouse CD8a (BD Pharmingen, USA), PE-Cy5-conjugated anti-mouse CD25 (eBioscience, USA), FITC-conjugated mouse IgG2b, κ (BD Pharmingen, USA), FITC-conjugated rat IgG2a, κ (eBioscience, USA), PE-conjugated rat IgG2a, κ (eBioscience, USA), PE-conjugated mouse IgG1, κ (BD Pharmingen, USA). Cells were resuspended in FACS buffer to a concentration of 2 × 10^7^/ml and pre-incubated with 1 μg of anti-mouse CD16/CD32 (eBioscience, USA) per million cells for 5 min on ice prior to staining. Primary antibodies were diluted to predetermined optimal concentrations in 50 μl of FACS buffer and dispensed into each well of a 96-well microtiter plate. 50 μl of cell suspension was added to each well and incubated for 20 min at 4°C in the dark. After staining, cells were washed twice and resuspended in 100 μl FACS buffer. Intracellular staining of mouse Foxp3 was performed using PE anti-mouse Foxp3 Staining Set (eBioscience) according to the manufacturer's protocol. The sample data were acquired on a FACSCalibur flow cytometer (Becton Dickinson, USA) and analyzed with WinMDI software (Joseph Trotter).

### *In vitro *cytokine stimulation

Spleen cells were cultured at 2 × 10^6^/ml in a 96-well flat-bottom culture plate in complete RPMI-1640 medium. The cells were stimulated with 100 ng/ml LPS (Ultra-Pure LPS, Invivogen, USA) and 1.5 μg/ml ConA (Sigma-Aldrich, USA) and incubated in a 5% CO_2 _at 37°C for 48 h. Culture supernatants were collected after 48 h and stored at -20°C until analysis. Cytokine profiles were determined using a multiplex cytokine analyzer (Luminex).

### CFSE proliferation assay

Freshly isolated lymphocytes were resuspended in the solution of carboxyfluorescein diacetate succinimidyl ester (CFSE, 5 μM final concentration) and mixed rapidly. After 5 min at room temperature, the cells were washed three times with 10 volumes of PBS containing 5% FBS. CFSE-labeled cells were stimulated with 1.5 μg/ml ConA (Sigma-Aldrich, USA) in 96-well plates for 48 h, and then CFSE dilution was analyzed by flow cytometry.

### Multiplex cytokine determination

To determine cytokine profiles in culture supernatants we used the Antibody Bead Kits (BioSource, USA), which are designed to be analyzed with the Luminex^® ^200™ System (Luminex Corporation, USA). The samples were stained and analyzed according to the manufacturer's recommendations. Briefly, beads of defined spectral properties conjugated to analyte-specific capture antibodies and samples were pipetted into the wells of a filter bottom microplate (Millipore, USA) and incubated for 2 h. After washing the beads, analyte-specific biotinylated detector antibodies were added and incubated with the beads for 1 h. After removal of excess biotinylated detector antibodies, streptavidin conjugated to R-Phycoerythrin was added for 30 min. After washing, the beads were analyzed with the Luminex 200™ instrument. The concentrations of analytes were determined by monitoring the spectral properties of the beads and the amount of R-Phycoerythrin fluorescence.

### Data analysis

Analysis of data was conducted using BioStat 2007 software (AnalystSoft, Vancouver, CA). Data are expressed as mean ± standard error (SE). Differences between groups were examined for statistical significance using one-way analysis of variance (ANOVA) with a post-hoc analysis (Tukey-Kramer test). Values of *p *< 0.05 were regarded as significant and are denoted in the figures. * indicates p < 0.05 and ** indicates p < 0.01.

## List of abbreviations

LPS: lipopolysaccharide; GF: germ-free; CV: conventional; GALT: gut-associated lymphoid tissue; AIN-93G: a purified diet (low LPS diet); ST1: a grain-based diet (LPS-rich diet); Tregs: regulatory T cells; DCs: dendritic cells; MAMPs: microbe-associated molecular patterns; TLR: Toll-like receptor; CLR: C-type lectin receptor; NLR: Nod-like receptor; MLNs: mesenteric lymph nodes; PPs: Peyer's patches; PC: peritoneal cells; CFSE: carboxyfluorescein diacetate, succinimidyl ester; ConA: concanavalin A.

## Authors' contributions

TH carried out the whole studies and drafted the manuscript. RS participated in the design of the study and rederived Balb/c mice in germ-free conditions. HTH supervised the study, and participated in its design and coordination and helped to draft the manuscript. All authors read and approved the final manuscript.

## Supplementary Material

Additional file 1 **Table D1. **Composition of semi-purified diet (AIN-93G). Click here for file

Additional file 2 **Table D2. Composition of grain-based diet (ST1).** Click here for file
