# Supplementary material for: Gut microbiota and lipopolysaccharide content of the diet influence development of regulatory T cells: studies in germ-free mice
Source: BMC Immunol. 2008 Nov 6;9:65. doi: 10.1186/1471-2172-9-65 (PMC2588440; doi:10.1186/1471-2172-9-65)
Supplement: Additional file 1 — Table D1. Composition of semi-purified diet (AIN-93G). [file 1471-2172-9-65-S1.pdf]

AIN-93G Growth Semi-Purified Diet 5801-G*

DESCRIPTION

AIN-93G is the growth diet for rodents recommended by the American Institute of Nutrition. It is formulated to substitute for the previous version (AIN-76A) to improve animal performance.

TYPICAL ANALYSIS

Protein	18.7%
Fat	7.0%
Fiber	5.0%
Carbohydrate	64.7%
Metabolizable Energy, kcal/gm	3.97

FEEDING DIRECTIONS

Feed ad libitum to mice and rats. Plenty of fresh, clean water should be available at all times.

INGREDIENTS

INGREDIENTS	g/kg diet
Corn Starch	397.486
Casein (≥85% protein)	200.000
Dextrinized Corn Starch	132.000
Sucrose	100.000
Soybean Oil (no additives)	70.000
Fiber	50.000
Mineral Mix (AIN-93G-MX)	35.000
Vitamin Mix (AIN-93-VM)	10.000
L-Cystine	3.000
Choline Bitartrate (41.1% choline)	2.500
Tert-butylhydroquinone	0.014

AIN-93G-MX Mineral Mix	*g/kg mix*
Calcium Carbonate, Anhydrous, 40.04% Ca	357.00
Potassium Phosphate, Monobasic, 22.76% P; 28.73% K	196.00
Potassium Citrate Tri Potassium, Monohydrate, 36.16% K	70.78
Sodium Chloride, 39.34% Na; 60.66% Cl	74.00
Potassium Sulfate, 44.87% K; 18.39% S	46.60
Magnesium Oxide, 60.32% Mg	24.00
Ferric Citrate, 16.5% Fe	6.06
Zinc Carbonate, 52.14% Zn	1.65
Manganous Carbonate, 47.79% Mn	0.63
Cupric Carbonate, 57.47% Cu	0.30
Potassium Iodate, 59.3% I	0.01
Sodium Selenate, Anhydrous, 41.79% Se	0.01025
Ammonium Paramolybdate, 4 Hydrate, 54.34% Mo	0.00795
Potentially beneficial mineral element	
Sodium Meta-silicate, 9 Hydrate, 9.88% Si	1.45
Chromium Potassium Sulfate, 12 Hydrate, 10.42% Cr	0.275
Lithium Chloride, 16.38% Li	0.0174
Boric Acid, 17.5% B	0.0815
Sodium Fluoride, 45.24% F	0.0635
Nickel Carbonate, 45% Ni	0.0318
Ammonium Vanadate, 43.55% V	0.0066
Powdered Sucrose	221.026

AIN-93-VX Vitamin Mix	*g/kg mix*
Nicotinic Acid	3.000
Ca Pantothenate	1.600
Pyridoxine-HCL	0.700
Thiamin-HCL	0.600
Riboflavin	0.600
Folic Acid	0.200
D-Biotin	0.020
Vitamin B-12 (cyanocobalamin) (0.1% in mannitol)	2.500
Vitamin E (all-rac-α-tocopheryl acetate) (500 IU/g)	15.000
Vitamin A (all-trans-retinyl palmitate) (500,000 IU/g)	0.800
Vitamin D3 (cholecalciferol) (400,000 IU/g)	0.250
Vitamin K (phylloquinone)	0.075
Powdered Sucrose	974.655

*Product Code

CHEMICAL COMPOSITION[1]

Nutrients[2]	
**Protein, %**	**17.9**
Arginine, %	0.64
Cystine, %	0.37
Glycine, %	0.32
Histidine, %	0.46
Isoleucine, %	0.85
Leucine, %	1.54
Lysine, %	1.30
Methionine, %	0.46
Phenylalanine, %	0.88
Tyrosine, %	0.93
Threonine, %	0.67
Tryptophan, %	0.21
Valine, %	1.00
**Fat, %**	**7.0**
Cholesterol, ppm	0
**Fiber (Crude), %**	**5.0**
**Carbohydrate, %**	**64.4**
**Energy** (Physiological Fuel Value[3]), **kcal/gm**	**3.77**

Minerals	
**Ash, %**	**4.2**
Calcium, %	0.50
Phosphorus, %	0.30
Potassium, %	0.36
Magnesium, %	0.05
Sulfur, %	0.17
Sodium, %	0.10
Chlorine, %	0.16
Fluorine, ppm	1.0
Iron, ppm	45
Zinc, ppm	38
Manganese, ppm	10
Copper, ppm	6.0
Iodine, ppm	0.20
Chromium, ppm	1.0
Molybdenum, ppm	0.15
Selenium, ppm	0.18

Vitamins	
Vitamin K (as menadione), ppm	0.09
Thiamin Hydrochloride, ppm	5.0
Riboflavin, ppm	6.0
Niacin, ppm	30
Pantothenic Acid, ppm	15
Choline Chloride, ppm	1000
Folic Acid, ppm	2.0
Pyridoxine, ppm	6.0
Biotin, ppm	0.20
Vitamin B_{12}, mcg/kg	25
Vitamin A, IU/gm	4.0
Vitamin D_3 (added), IU/gm	1.0
Vitamin E, IU/kg	75

Calories provided by:	
Protein, %	19.300
Fat (ether extract), %	16.700
Carbohydrates, %	64.000

*Product Code

1. Based on the latest ingredient analysis information. Since nutrient composition of natural ingredients varies, analysis will differ accordingly.
2. Nutrients expressed as percent of ration except where otherwise indicated.
3. Physiological Fuel Value (kcal/gm) = Sum of decimal fractions of protein, fat and carbohydrate (use Nitrogen Free Extract) x 4,9,4 kcal/gm respectively.
