# Supplementary material for: Gut microbiota and lipopolysaccharide content of the diet influence development of regulatory T cells: studies in germ-free mice
Source: BMC Immunol. 2008 Nov 6;9:65. doi: 10.1186/1471-2172-9-65 (PMC2588440; doi:10.1186/1471-2172-9-65)
Supplement: Additional file 2 — Table D2. Composition of grain-based diet (ST1). [file 1471-2172-9-65-S2.pdf]

Additional file 2: Table D2

Composition of grain-based diet (ST1)

Ingredients - Ground wheat, ground oats, ground corn, wheat meal, dehydrated alfalfa meal, soybean meal, fish meal, feeding calcite, dicalcium phospate, feeding salt, methionin, vitamins.

Average nutrient composition		
Protein	%	24.00
Fat	%	3.72
Fiber	%	3.47
Ash	%	6.51
Minerals		
Calcium	%	1.32
Phosphorus	%	0.87
Natrium	%	0.18
Copper	mg/kg	22.60
Selenium	mg/kg	0.34
Vitamins		
Vitamin A	IU/kg	332.00
Vitamin D3	IU/kg	2.50
Vitamin E	mg/kg	107.90
